# Identification and First Report of *Fusarium andiyazi* Causing Sheath Rot of *Zizania latifolia* in China

**DOI:** 10.3390/plants10091844

**Published:** 2021-09-06

**Authors:** Ya-Min Ma, Jun-Zi Zhu, Xiao-Gang Li, Lai-Liang Wang, Jie Zhong

**Affiliations:** 1 Hunan Provincial Key Laboratory for Biology and Control of Plant Diseases and Insect Pests, Hunan Agricultural University, Nongda Road 1, Furong District, Changsha 410128, China; mym13957049350@sina.com (Y.-M.M.); zjz0808@gmail.com (J.-Z.Z.); 2 Jinyun Plant Protective Station, Daqiao North Road 290, Lishui 321400, China; 3 Hunan Engineering Research Center of Agricultural Pest Early Warning and Control, Hunan Agricultural University, Nongda Road 1, Changsha 410128, China; 4 Lishui Institute of Agricultural and Forestry Sciences, Liyang Stress 827, Lishui 323000, China

**Keywords:** *Fusarium andiyazi*, morphological characterization, jiaobai, phylogenetic analysis

## Abstract

*Zizania latifolia* is a perennial plant native to East Asia. The swollen culm of *Z. latifolia* is a popular vegetable and traditional herbal medicine consumed in China and some other Asian countries. From 2019 to 2021, a sheath rot disease was found in Zhejiang Province of China. Symptoms mainly occurred in the leaf sheath showing as brown necrotic lesions surrounded by yellow halos. The pathogen fungal isolates were isolated from the affected sheaths. Ten representative isolates were selected for morphological and molecular identification by phylogenetic analyses of the translation elongation factor 1-α (*TEF1*) and the RNA polymerase II subunit beta (*RPB2*) gene regions. Based on the combined datasets, the fungal isolates were identified as *Fusarium andiyazi*. Koch’s postulates were confirmed by pathogenicity test, re-isolation and re-identification of the fungal isolates. To the best of our knowledge, this is the first report of sheath rot caused by *F. andiyazi* in *Z. latifolia* in China.

## 1. Introduction

*Zizania latifolia* belongs to the rice tribe (Oryzeae) of the grass family Poaceae [1], also called jiaobai in China, due to its white bamboo [2]. *Z. latifolia* is a perennial aquatic herbaceous plant. Its terrestrial stem can from 2 to 3 tilers. Due to its unique taste and nutritional and economic importance, *Z. latifolia* has been domesticated as a vegetable crop for approximately 2000 years and has become a delicacy for people’s daily consumption. It is widely cultivated in China, Japan, Korea and countries in Southeast Asia [3]. The Jiangsu and Zhejiang provinces of China have the largest cultivation areas of this plant [4]. 

A few diseases have been found to be associated with *Z. latifolia*, thus limiting the production of this plant. Several pathogenic fungi have been found in *Z. latifolia*, including *Bipolaris* [5], *Claviceps* [6], *Pyricularia* [7] and *Pythiogeton* [7,8]. However, up to now, no *Fusarium* spp. has been reported to cause disease in *Z. latifolia.*

Sheath rot is one of the most severe diseases in crops, and prevalence of the disease can lead to severe crop loss. In the natural state of the field, the disease mainly appears in leaf sheaths and forms irregular, brown, erosive spots, so it is called sheath rot. The spots first were water-stained elliptic or brown dots, and then they expanded. Multiple spots were converged to form black and brown irregular patches, which gradually spread to the lower or upper parts of the leaf sheaths and resulted in the entire sheath to wither and die. Corn *Fusarium* sheath rot (CFSR) is a serious crop disease in China that has been caused by Fusarium species, such as *F. graminearum*, *F. verticillioides*, *F. equiseti*, *F. fujikuroi*, *F. meridionale* and *F. asiaticum* [9,10]. Therefore, according to symptoms and pathogens, we named the disease identified in this study sheath rot.

According to previous studies, *Fusarium* spp. are important plant pathogens and can cause many diseases in plants of the grass family *Poaceae*, such as ear rot, stalk rot, seedling blight and root rot in maize [11,12]. It is difficult to distinguish *Fusarium* spp. by only morphology because of the variety of species [13]. Therefore, molecular-based techniques were developed and became simple, rapid and efficient methods for identification of fungal species [14]. *Fusarium andiyazi* has been found in some plants, such as maize [15], sorghum [16] and rice [17,18]. However, it has not been found in *Z. latifolia.*

The purpose of this study was to isolate and identify the causal pathogen of a novel sheath rot disease of *Z. latifolia*. Conventional morphological identification, together with DNA sequencing of parts of the translation elongation factor-1a (*TEF1*) and RNA polymerase II subunit beta (*RPB2*) genes, was carried out. This research will provide an important basis for effective control strategies for this disease.

## 2. Results

### 2.1. Occurrence of Sheath Rot in Zizania latifolia

**Figure 1:**
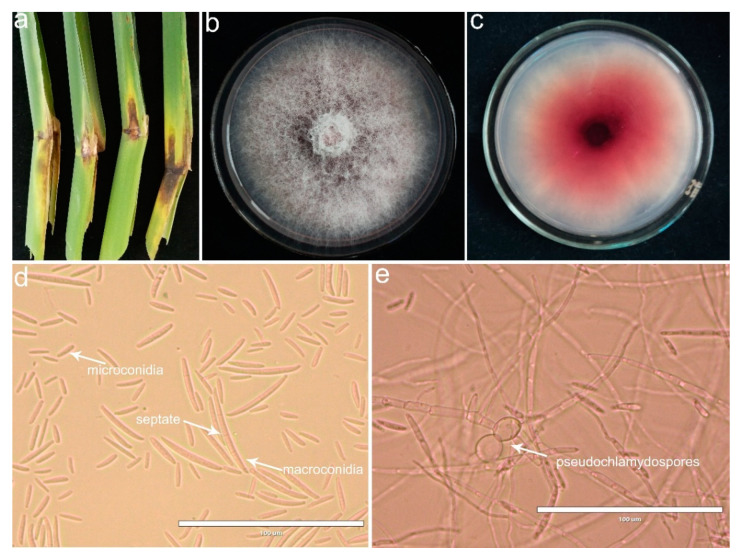
Symptoms of sheath rot observed on *Zizania latifolia* and morphological and cultural characteristics of colonies and conidia. (**a**) Sheath rot in field-collected *Z. latifolia* plants; (**b**) positive sides of the colonies cultured on potato dextrose agar (PDA) for 6 days; (**c**) reverse sides on PDA; (**d**) micrographs of the microconidia and the macroconidia; (**e**) micrographs of the pseudochlamydospores. Scale bar = 100 μm.

From 2019 to 2021, a disease of sheath rot was observed in *Z. latifolia* in Zhejiang Province of China. Disease symptoms mainly occurred in the leaf sheath showing as water-soaking chlorosis spots on the ear leaf near the sheath in the early stage, then expanded and extended to the lower leaf sheath surrounded by yellow halos, with a diffusion diameter of more than 5 to 10 cm. At a later stage, lesions developed into a brown to black color and had a white mycelium layer faintly visible in the lesions (Figure 1a,b). Among twenty-eight investigated wild fields, the disease incidence ranged from 30% to 80%, with an average of 56.71 ± 2.59%.

### 2.2. Fungal Isolation and Morphological Identification

A total of fifty-eight fungal isolates were obtained from sixty *Z. latifolia* sheath samples collected from Lishui, Jinhua, Taizhou and Jiaxing of the Zhejiang Province of China. Based on the morphology observation, these fungal isolates were putatively known as *Fusarium* species. We selected ten isolates for further morphological and molecular identification. When cultured on PDA medium, the fungal isolates were white and gradually varied from tan to pale lilac, exhibiting floccose to powdery mycelium. Fungal isolates produced abundant conidia after 7 days of culture on carnation leaf agar (CLA). Microscopic observations revealed that the macroconidia were straight or slightly curved, had a pedicellate basal cell and slightly curved apical cell, with 3–5 septate, and measured 30.99 − 80.95 × 1.54 − 4.63 mm. Microconidia were unicellular, oval with a flat base, aseptate, measuring 6.85 − 24.81 × 1.17 − 4.68 mm in size. Pseudochlamydospores with smooth thinner walls and in solitary or short chains were observed in hyphae. The features of the microconidia and macroconidia are described in Table 1.

### 2.3. Molecular Identification and Phylogenetic Analysis

**Figure 2:**
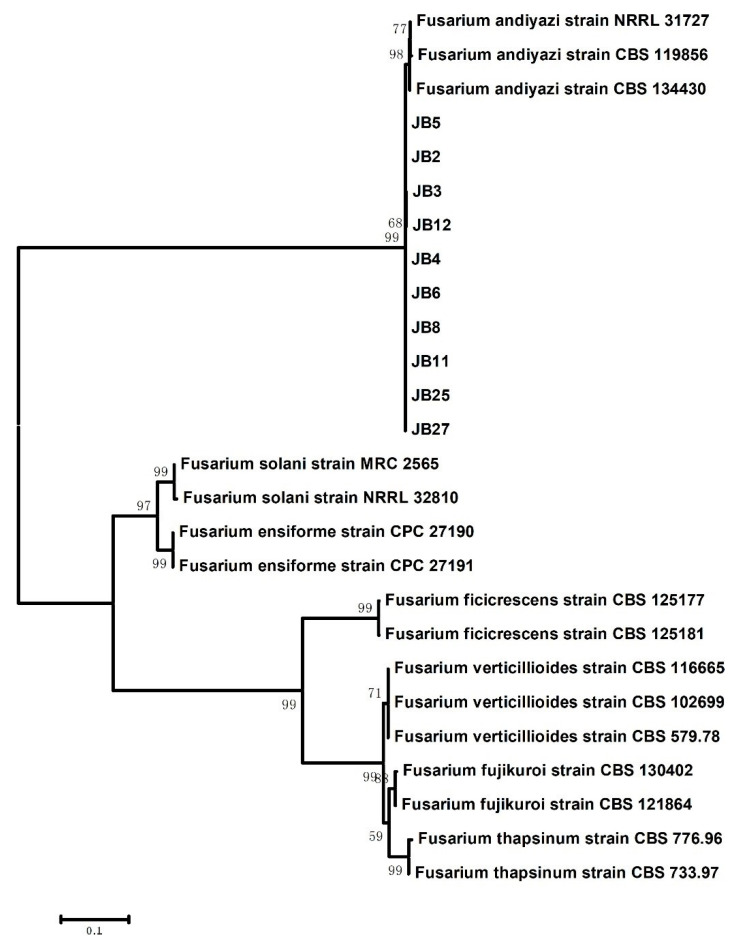
Phylogeny of the representative isolates from *Z. latifolia* and other *Fusarium* species. Phylogenetic tree was constructed based on concatenated sequences of translation elongation factor 1-α (*TEF1*) and the RNA polymerase II subunit beta (*RPB2*) gene regions. Numbers in the branch were calculated from the bootstrap test of 1000 replicates. Information of the fungal isolates used for phylogenetic tree construction are shown in Table 2.

For further molecular verification, the *TEF1* and *RPB2* gene sequences of ten representative isolates were amplified and sequenced. All of the obtained sequences were submitted to the NCBI database under the accession numbers listed in Table 2. After analyzing with BLASTn against the NCBI (http://www.ncbi.nlm.nih.gov, accessed on 22 June 2021) and FUSARIUM ID (http://isolate.fusariumdb.org/guide.php, accessed on 22 June 2021) databases, these obtained *TEF1* sequences were 99.23% to 99.54% identical to sequences of *F. andiyazi* strains and had 98.34% to 98.64% identity with *F. andiyazi* strains, such as FD_01386_EF-1a, in Fusarium ID. For *RPB2*, these isolates also showed 99.05% to 99.37% identity with *F. andiyazi* strains in GenBank. The ten representative isolates and a total of 17 other *Fusarium* isolates were selected for phylogenetic analysis using the concatenated *TEF1* and *RPB2* gene sequences (Figure 2). Phylogenetic tree analysis revealed that our ten representative isolates were clustered with the *F. andiyazi* clade, including *F. andiyazi* strains NRRL 31727, CBS 119856 and CBS 134430 based on the neighbor-joining (NJ) method, which was consistent with the BLASTn homology search results.

### 2.4. Pathogenicity Assays

**Figure 3:**
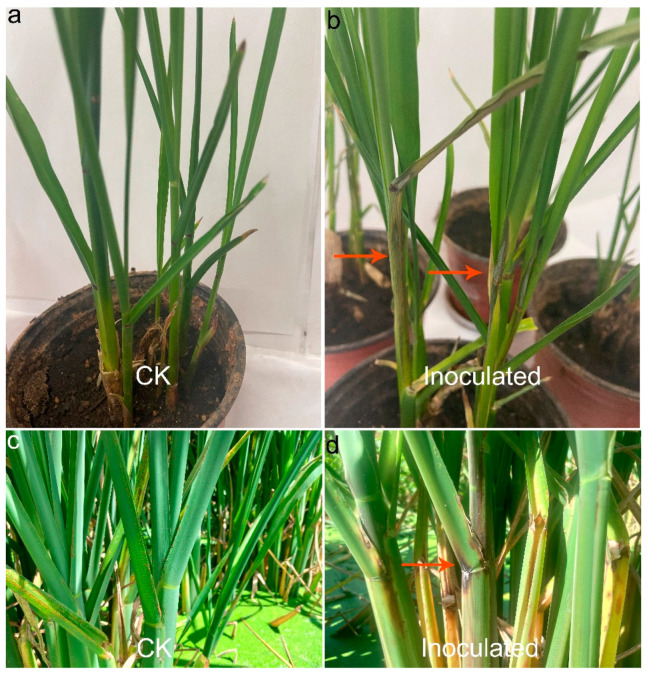
Pathogenicity test on *Z. latifolia* leaf sheaths. (**a**,**c**) Control inoculated plants of potted and field *Z. latifolia* plants, respectively; (**b**,**d**) symptoms of water-soaking and brown necrotic patches were observed on the ear leaf near the sheath of potted and field *Z. latifolia* plants after three and four days of inoculation, respectively. No obvious symptoms developed on the control plants.

To assess the pathogenicity of the isolated *F. andiyazi* isolates, in vivo inoculation experiments with conidial suspensions were conducted in potted and field *Z. latifolia* plants (Figure 3). Four representative isolates JB-2, JB-5, JB-6 and JB-27 were randomly selected. Three days after inoculation, symptoms of water soaking were observed on the ear leaf near the sheath. As the disease progressed, the lesions turned visibly dry and brown. In the field inoculation experiment, symptoms displaying as water-soaking and dark brown necrotic lesions surrounded by yellow halos were observed on the inoculated plants after 4 to 5 days of inoculation. No symptoms developed on the control plants. The pathogen fungal isolates were re-isolated from the symptomatic inoculated leaf sheath of *Z. latifolia*, and their identities were confirmed by morphological and molecular methods as described above, thus fulfilling Koch’s postulates. This was the first report of *F. andiyazi* causing sheath rot in *Z. latifolia*.

## 3. Discussion

In this study, according to cultural and conidial morphology, *TEF1-* and *RPB2*-sequence-based phylogenetic analysis and pathogenicity tests, the pathogen fungus was identified as *F. andiyazi* and confirmed to be the causal agent of sheath rot in *Z. latifolia*. Therefore, this study provided the first evidence that *F. andiyazi* is responsible for sheath rot of *Z. latifolia.* The identification of the pathogen will provide important insights for appropriate field management and control of this new disease. 

*Fusarium andiyazi* was first reported in sorghum (*Sorghum bicolor* L.) in Africa and the United States [19] and was considered one of the most virulent species causing rot and stalk rot on sorghum seedlings [20,21,22]. Up to date, *F. andiyazi* has been reported to cause disease on many plants worldwide, such as pokkah boeng on sugarcane [23,24,25], ear and root rot in maize [26,27], vascular wilts in oriental melons [28], wilting disease in tomatoes [29], Bakanae on rice [16,18,30,31] and seedling wilt and root rot on sugar beets [32]. However, there are no reports of *F. andiyazi* causing disease in *Z. latifolia*.

In this study, we identified the isolated fungal isolates as *F. andiyazi* based on both morphological characteristics and molecular sequencing. Because the ITS loci are too conserved to resolve species limits of most fusaria, ITS-rDNA sequencing was not sufficient for differentiating closely related *Fusarium* spp. The *TEF1* and *RPB2* sequences were proven to be reasonable loci for identification of *Fusarium* spp. [33]. In our study, the *TEF1* and *RPB2* sequences of ten representative isolates were sequenced and blasted against the NCBI and FUSARIUM ID databases. Phylogenetic analysis with the combined *TEF1* and *RPB2* sequences revealed that our isolated isolates were clustered in the *F. andiyazi* clade, which was consistent with morphological observations. 

*Zizania latifolia* is a perennial plant native to Asia and is widely grown in China and other Asian countries as a popular aquatic vegetable [4,34]. In addition, the swollen culm of *Z. latifolia*, also known as Jiaobai or Gaogua in China, was recorded as traditional Chinese herbal medicine used for heat clearing, detoxifying, quenching thirst, diuresis, etc. [35]. With food and medicinal values, the cultivation area of *Z. latifolia* has been largely expanded in China, especially south of the Yangtze River. In this study, sheath rot disease occurred with a high incidence, which damaged the sheath plant, leading to serious loss of production. Its occurrence might bring about a threat to *Z. latifolia*. Thus, further research for effective management strategies should be conducted to reduce the damage caused by this pathogen.

In conclusion, we identified the causal agent of a novel sheath rot of *Z. latifolia*. Morphological characteristics of the fungal isolates were observed on the artificial culture medium. Gene sequencing of the *TEF1* and *RPB2* genes was conducted and confirmed the pathogen to be *F. andiyazi*. Pathogenicity of the isolates was tested, and a difference between isolates was shown. The disease was found to be prevalent in Zhejiang Province of China. However, further research of the distribution of *F. andiyazi* on other areas, genetic diversity and pathogenicity differentiation of the pathogen are needed. 

## 4. Materials and Methods

### 4.1. Sample Collection and Isolation

*Z. latifolia* plants infected with visible necrosis were collected from different fields in Lishui, Jinhua, Taizhou and Jiaxing of Zhejiang Province, China. Diseased sheaths were characterized by black-brown lesions surrounded by yellow halos. Two diseased leaf sheaths were collected from survey spots and subjected to pathogen isolation. Symptomatic tissues, in approximately 5 × 5 mm fragments, were cut from the edge of lesions, surface sterilized with 70% ethanol for 30 s and 1% NaClO for 1 min, washed with sterile distilled water three times and then placed onto PDA supplemented with 100 mg/mL of streptomycin. After 2–3 days of incubation at 26 °C in the dark, emerged hyphal tips were picked and transferred onto fresh PDA and kept at 26 °C. Fungal cultures were further purified by the single-spore isolation technique [36] and stored at 4 °C in a refrigerator. 

### 4.2. Morphological and Cultural Characterization

For morphological examination, mycelial plugs (8 mm diam) of the fungal isolates were taken from actively growing areas of 5-day-old cultures and plated on PDA at 26 °C for 6 to 7 days. Colony characteristics, including morphology and color, were observed after 7 days of cultivation. Conidial production was induced after the fungal isolates were grown on CLA at 26 °C for 7 days, which was the most suitable medium for *Fusarium* identification, as typical macroconidia, microconidia and chlamydospores were consistently produced in this medium [37]. The morphology of the conidia was examined and photographed under an optical microscope (Life Technologies, EVOS™ XL Core Imaging System), and their length and width were determined by measuring about 50 randomly selected conidia.

### 4.3. DNA Extraction, PCR Amplification, and Sequencing

Genomic DNA of ten representative fungal isolates was extracted using the CTAB method as described previously [38]. Mycelium was harvested from the colony surface by a sterile medicine spoon. The *TEF1* and *RPB2* genes were amplified using the primer pairs EF-1/EF-2 (5′-ATGGGTAAGGARGACAAGAC-3′/5′-GGARGTACCAGTSATCAT G-3′) and 7cf/11ar (5′-CCCATRGCTTGYTTRCCCAT-3′/5′-GCRTGGATCTTRTCRTCSA CC-3′) [39,40]. Polymerase chain reaction (PCR) amplification was conducted in 50 μL volume reaction system containing 25 μL of 2 × PCR Master Mix (Vazyme, Nanjing, China), 2 μL of each primer (10 μM), 2 μL of genomic DNA template and 19 μL of sterile distilled water. PCR was performed with the thermal cycling parameters of 95 °C for 3 min, followed by 34 cycles of denaturation at 95 °C for 30 s, annealing at 54–57 °C for 30 s, extension at 72 °C for 1 min and final extension at 72 °C for 8 min. The PCR products were visualized by running with 1.0% agarose gel and staining with GoldView™. The PCR products were sequenced using the dideoxy termination method in Shanghai Sangon Company in China. 

### 4.4. Phylogenetic Analysis

All the generated sequences were blasted against the NCBI database and the FUSARIUM ID database for homology searching. Sequences of the top matches and other ex-type isolates of *Fusarium* species were selected and downloaded from the database for phylogenetic analyses. Sequences were aligned with ClustalX [41], and phylogenetic analysis was performed using the MEGA6 software package [42]. A concatenated phylogenetic tree based on the *TEF* and *RPB1* sequences was constructed using the NJ approach, with the bootstrap values calculated by 1000 replications.

### 4.5. Pathogenicity Tests

Pathogenicity test was conducted by in vivo inoculation with potted and field *Z. latifolia* plants with conidial suspensions. Four isolates were selected for pathogenicity assays that were cultured on PD liquid medium at 26 °C for 4 days in a shaker incubator (26 °C, 180 rpm/min). Conidial suspensions were harvested by filtering with four layers of a sterile gauze and then adjusted to a final concentration of 1 × 10^5^ spores/mL by a hemocytometer. *Z. latifolia* plants were transplanted in pots that were placed in a greenhouse for one month, and field plot contained 20 lines and 10 plants per line. For greenhouse inoculation, a 1 mL aliquot of conidial suspensions for the four fungal isolates was injected into the ear leaf near to sheath in a total of fifteen *Z. latifolia* seedlings planted in three pots, while sterile water was inoculated to fifteen control plants with the same procedure. All the inoculated plants were coated with plastic bags and maintained in a humid growth chamber at 26 °C under a 16/8 h light/dark cycle. The progression of symptoms was observed daily until necrotic lesions formed. For the field inoculation experiment, a 2 mL aliquot of the conidial suspensions was injected into the ear leaf as described above. The control plants were inoculated with sterile water. All the inoculated sites were covered with sterile cotton wet with sterile water to maintain moisture. After five to seven days, disease symptoms were assessed. The fungal isolates were considered pathogenic if brown or black necrotic lesions occurred in the inoculated sites. Fungal isolates were re-isolated from the symptomatic inoculated sheaths and re-identified with morphological and molecular characteristics to confirm Koch’s postulates.

### 4.6. Data Analysis

Statistical analyses were conducted using Statistical Package for Social Sciences (SPSS) (version 22.0 for Windows). Analysis of variance (ANOVA) in the conidial length and width were performed. Means were compared by the least significant difference test at a significance level of *p* = 0.05.

## Figures and Tables

**Table 1:** Micromorphology of the microconidia and macroconidia of ten representative isolates produced on carnation leaf agar medium.

Isolate	Conidial Size (μm) ^a^				Mean ± SD ^b^ (μm)	
	Microconidia		Macroconidia		Microconidia	Macroconidia
	Length	Width	Length	Width		
JB-2	7.28–24.81 ^a^	1.32–4.30 ^a^	32.48–80.47 ^a^	1.60–4.63 ^a^	14.50 ± 0.69 × 2.68 ± 0.12	44.37 ± 1.48 × 3.35 ± 0.12
JB-3	7.13–24.56 ^a^	1.23–4.02 ^a^	31.95–78.16 ^a^	1.69–4.37 ^a^	14.56 ± 0.60 × 2.69 ± 0.10	43.97 ± 1.72 × 3.19 ± 0.11
JB-4	6.85–25.29 ^a^	1.38–4.14 ^a^	31.36–81.35 ^a^	1.54–4.47 ^a^	14.55 ± 0.61 × 2.53 ± 0.09	43.96 ± 1.69 × 3.25 ± 0.10
JB-5	7.43–24.67 ^a^	1.17–4.55 ^a^	31.64–79.51 ^a^	1.82–4.55 ^a^	14.74 ± 0.58 × 2.7 ± 0.11	44.31 ± 1.62 × 3.03 ± 0.09
JB-6	7.55–22.92 ^a^	1.51–3.91 ^a^	32.55–80.76 ^a^	1.96–4.60 ^a^	14.18 ± 0.45 × 2.58 ± 0.09	44.83 ± 1.53 × 3.30 ± 0.11
JB-8	7.97–23.45 ^a^	1.15–4.60 ^a^	31.82–81.09 ^a^	2.02–4.32 ^a^	14.44 ± 0.62 × 2.70 ± 0.13	44.16 ± 1.74 × 3.01 ± 0.10
JB-11	7.21–23.78 ^a^	1.37–4.16 ^a^	30.99–80.95 ^a^	1.88–4.09 ^a^	14.63 ± 0.56 × 2.74 ± 0.11	43.81 ± 1.60 × 2.99 ± 0.09
JB-12	7.89–24.35 ^a^	1.25–4.68 ^a^	32.03–80.28 ^a^	2.27–4.14 ^a^	14.75 ± 0.55 × 2.71 ± 0.12	44.32 ± 1.39 × 3.19 ± 0.07
JB-25	7.76–24.03 ^a^	1.68–3.97 ^a^	31.74–80.19 ^a^	2.11–4.26 ^a^	14.38 ± 0.56 × 2.67 ± 0.08	44.04 ± 1.31 × 3.02 ± 0.08
JB-27	7.46–23.86 ^a^	1.43–4.09 ^a^	31.58–79.93 ^a^	2.05–4.21 ^a^	14.46 ± 0.58 × 2.57 ± 0.10	44.56 ± 1.62 × 3.21 ± 0.09

^a^ Numbers indicate minimum and maximum lengths and widths, respectively, of 50 conidia recorded from each fungal isolate. Significance at *p* = 0.05 level. ^b^ SD means standard deviation. Values within the same column followed by the same letters mean that they are not significantly different based on variance with least significant difference test at *p* = 0.05.

**Table 2:** GenBank accession numbers of the *Fusarium* isolates used in this study for phylogenetic analysis.

Species	Isolate/Strain	GenBank Accession Number	
		*TEF1*	*RPB2*
*Fusarium andiyazi*	NRRL 31727	MN193854.1	MN193882.1
*Fusarium andiyazi*	CBS 119856	MN533989.1	MN534286.1
*Fusarium andiyazi*	CBS 134430	KC954401.1	LR792614.1
*Fusarium andiyazi*	JB-2	MZ396373	MZ396383
*Fusarium andiyazi*	JB-3	MZ396374	MZ396384
*Fusarium andiyazi*	JB-4	MZ396375	MZ396385
*Fusarium andiyazi*	JB-5	MZ396376	MZ396386
*Fusarium andiyazi*	JB-6	MZ396377	MZ396387
*Fusarium andiyazi*	JB-8	MZ396378	MZ396388
*Fusarium andiyazi*	JB-11	MZ396379	MZ396389
*Fusarium andiyazi*	JB-12	MZ396380	MZ396390
*Fusarium andiyazi*	JB-25	MZ396381	MZ396391
*Fusarium andiyazi*	JB-27	MZ396381	MZ396392
*Fusarium solani*	MRC 2565	MH582420.1	MH582410.1
*Fusarium solani*	NRRL 32810	DQ247118.1	EU329624.1
*Fusarium ensiforme*	CPC 27190	LT746199.1	LT746312.1
*Fusarium ensiforme*	CPC 27191	LT746200.1	LT746313.1
*Fusarium ficicrescens*	CBS 125177	KP662898.1	KT154001.1
*Fusarium ficicrescens*	CBS 125181	KP662900.1	KT154003.1
*Fusarium fujikuroi*	CBS 130402	KU604446.1	KU604261.1
*Fusarium fujikuroi*	CBS 121864	KU604442.1	KU604258.1
*Fusarium thapsinum*	CBS 733.97	KU604463.1	KU604299.1
*Fusarium thapsinum*	CBS 776.96	KU604462.1	KU604294.1
*Fusarium verticillioides*	CBS 102699	KU604385.1	KU604218.1
*Fusarium verticillioides*	CBS 579.78	KU604390.1	KU604223.1
*Fusarium verticillioides*	CBS 116665	KU604388.1	KU604221.1

## Data Availability

All sequence data are available in NCBI GenBank following the accession numbers in the manuscript.
